# *A De Novo* Mutation in *ACTC1* and a *TTN* Variant Linked to a Severe Sporadic Infant Dilated Cardiomyopathy Case

**DOI:** 10.1155/crig/9517735

**Published:** 2024-12-28

**Authors:** Jose G. Acuña-Ochoa, Norma A. Balderrábano-Saucedo, Ana C. Cepeda-Nieto, Maria Y. Alvarado-Cervantes, Vianca L. Ibarra-Garcia, Daniel Barr, Matthew J. Gage, Ryan Pfeiffer, Dan Hu, Hector Barajas-Martinez

**Affiliations:** ^1^Cardiovascular Research Department, Lankenau Institute for Medical Research, Lankenau Hearth Institute, Wynnewood, Pennsylvania 19096, USA; ^2^Cardiomyopathies and Arrhythmias Research Laboratory/Department, Federico Gómez Children's Hospital of Mexico, Mexico 06720, Mexico; ^3^Molecular Genomics Laboratory/Department, Faculty of Medicine, Universidad Autónoma de Coahuila, Saltillo, Coahuila 25000, Mexico; ^4^Therapeutic Innovation Program/Division, Center for Applied Medical Research, University of Navarra, Pamplona 31008, Spain; ^5^Chemistry Department, University of Mary, Bismarck, North Dakota 58504, USA; ^6^Chemistry Department, University of Massachusetts at Lowell, Lowell, Massachusetts, 01854, USA; ^7^Molecular Genetics Department, Masonic Medical Research Institute, Utica, New York 13501, USA; ^8^Department of Cardiology and Cardiovascular Research Institute, Renmin Hospital of Wuhan University, Wuhan, Hubei, China; ^9^Department of Pharmacology and Physiology, Sidney Kimmel Medical College, Thomas Jefferson University, Philadelphia, Pennsylvania, USA

**Keywords:** ACTC1, dilated cardiomyopathy, pathogenic variations, sudden cardiac death, TTN

## Abstract

Structural or electrophysiologic cardiac anomalies may compromise cardiac function, leading to sudden cardiac death (SCD). Genetic screening of families with severe cardiomyopathies underlines the role of genetic variations in cardiac-specific genes. The present study details the clinical and genetic characterization of a malignant dilated cardiomyopathy (DCM) case in a 1-year-old Mexican child who presented a severe left ventricular dilation and dysfunction that led to SCD. A total of 132 genes (48 structure- and 84 electrical-related genes) were examined by next generation sequencing to identify potential causative mutations in comparison to control population. *In silico* analysis identified only two deleterious heterozygous mutations within an evolutionarily well-conserved region of the sarcomeric genes *ACTC1*/cardiac actin (c.664G > A/p.Ala222Thr) and *TTN*/titin (c.33250G > A/p.Glu11084Lys). Further pedigree analysis revealed the father of the index case to carry with the *TTN* mutation. Surprisingly, the *ACTC1* mutation was not harbored by any first-degree family member. Computational 3D modeling of the mutated proteins showed electrostatic and conformational shifts of cardiac actin compared to wild-type version, as well as changes in the stability of the compact/folded states of titin that normally contributes to avoid mechanic damage. In conclusion, our findings suggest a likely pathogenic *de novo* mutation in *ACTC1* in coexpression of a *TTN* variant as possible causes of an early onset of a severe DCM and premature death. These results may increase the known clinical pathogenic variations that may critically alter the structure of the heart, whose fatality could be prevented when rapidly detected.

## 1. Introduction

Sudden cardiac death (SCD) is an unexpected lethal cessation of cardiac function that accounts for approximately half of the 17 million cardiovascular-related annual deceases that are estimated worldwide [1]. One of the significant causes of SCD is dilated cardiomyopathy (DCM), which is the leading global indication for heart transplantation (HT) [2]. DCM is defined as a condition in which the ventricles of the heart enlarge and the myocardium walls become thinner, compromising cardiac output and making it difficult to oxygenate the brain and body cells [3]. The pathological manifestations of DCM can vary from mild symptoms of heart failure (HF) in adults to the onset of SCD at an early age [4]. The prevalence of DCM in HF has been reported of approximately 1:250–400 and 5–7 new cases per 100,000 persons per year [5], although an accurate update estimation is difficult to assess.

Around 40% of familial DCM cases can be clarified by genetic variants, and most of them were inherited as an autosomal-dominant disease in families [4]. Diagnosis of SCD syndromes may be the most pressing challenge in cardiology due to its complex physiopathology, unknown molecular mechanisms and inadequacy of current genomic tools. Moreover, this challenge is compounded by difficulty in managing cardiac tissues. However, researchers continue to find new gene variants in many malignant cardiac cases such as DCM [6–8], that could be the cause of its etiology, which highlight the importance of genetic testing in certain individuals to implement an adequate preventive and personalized treatment plan if needed.

The current study describes the clinical features of a severe DCM in a 1-year-old infant and links this pathogenesis with the presence of two rare and presumptive deleterious genetic variants that change well evolutionary conserved regions in the *ACTC1* and *TTN* structural genes. Additionally, the *ACTC1* mutation appears to have a *de novo* origin, which highly supports its probable pathogenicity. Overall, the identification of these mutations broadens the base of pathogenic variants linked to SCD.

## 2. Methods

### 2.1. Subject and Methods

This study was approved by the Ethics, Biosafety, and Research Committees of the Children's Hospital of Mexico Federico Gómez, the Clinical Laboratory Evaluation Program from the New York State Department of Health, and by Main Line Health Institutional Review Board located at Lankenau Medical Center. Following the explanation of the study, the parents of the minor agreed to include him in the genetic screening, as well as themselves and the brother of the proband. The patient's clinical file, the laboratory database, and all the clinical images available in the hospital system were reviewed. The clinical information available includes familial and personal clinical history, all the clinical and surgical notes, physical examination notes, electrocardiogram results, thoracic roentgenography results, Doppler echocardiography results, along with the treatment indications and death certificate.

### 2.2. Genetic Study

Genomic DNA was extracted from peripheral blood lymphocytes using a commercial kit (Gentra System, Puregene, Valencia, California, the United States of America). The Supporting Tables S2 and S3 describe the library of 48 structure-related and 84 electrical-related genes, respectively, that were amplified using the Ion AmpliSeq Library Preparation protocol (Thermo Fisher Scientific, Waltham, Massachusetts, the United States of America). Clonal fragments were loaded into an Ion 318 Chip Kit v2 BC with the Ion Chef system and analyzed by NGS using the Ion Torrent Personal Genome Machine. After variant calling from the Ion Reporter software, genetic variants were further selected for identification of candidate deleterious mutations, selecting only those with MAF under 0.03, by comparison to the reference genomic DNA sequences from the 1000 genomes' project (https://www.ncbi.nlm.nih.gov/variation/tools/1000genomes/), as well as other previously described criteria [9], which include the score evaluation of each mutation according to the in silico prediction tests PolyPhen-2, PROVEAN, and SIFT.

### 2.3. Variant Validation

Genomic DNA (80–120 ng) was purified postamplification using the ExoSAP-IT kit (Applied Biosystems, Foster City, California, the United States of America), and sanger sequencing was performed with dideoxynucleotides using 0.8 μL of BigDye terminator v3.1 (Thermo Fisher Scientific, Waltham, Massachusetts, the United States of America) in the appropriate buffer, 0.5 *μ*M of each primer in separated reactions, 1 μL of PCR product, and 4 μL of HPLC grade water. Final PCR products were purified using Sephadex columns G-50 (Sigma-Aldrich, St. Louis, Missouri, the United States of America), and sequencing reactions were performed in an ABI PRISM 3100-Avant Genetic Analyzer (Applied Biosystems, Foster City, California, the United States of America). Electropherogram analysis of both DNA strands was achieved using sequencing analysis v.5.3 software (Applied Biosystems, Foster City, California, the United States of America).

### 2.4. Computational 3D Modeling Simulation and Protein Grade of Conservation

Protein structural homology of wild-type cardiac actin and its found variant were performed using the online workspace of SWISS-MODEL [10]. To generate the 3D protein structure, files from the protein data bank (PDB) were visualized in PyMOL 2.4.0 software (DeLano Scientific) [11], and the corresponding analysis of protein features was conducted. In parallel, the structure of wild-type cardiac titin and its variant were analyzed following the method of Cukier [12]. We used the probability of occupancy for each pair of amino acids in dihedral angle space to generate an ensemble of conformations of the peptide encompassing five residues flanking each side of the mutant. Each eleven-residue peptide had 1300 allowed conformations generated after removing the conformations with high-energy overlap of atoms that violate the Ramachandran map. The range of conformations and the end-to-end distance for each peptide was analyzed.

To assess the evolutionary importance of the amino acid change provoked by confirmed mutations, a comparison across different species was used to determine the amino acidic grade of conservation, wielding multiple protein alignment tools with the interest protein sequence from different species.

### 2.5. Heritability of Variants

After performing Sanger sequencing of the available proband's relatives for the confirmed variants, a pedigree tree was constructed to elucidate the heritability pattern.

## 3. Results

### 3.1. Clinical Case

**Figure 1:**
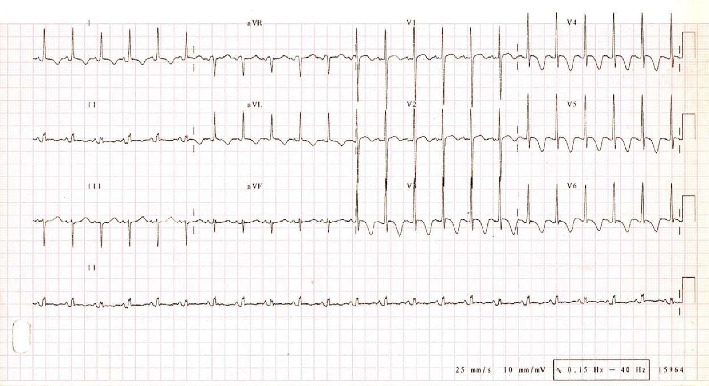
Electrocardiographic findings in the infant DCM patient. Twelve lead electrocardiogram shows sinus tachycardia of 159 bpm, increased precordial lead voltages, and an incomplete bundle brunch left ventricle (BBLV) and inverted *T* waves in V3–V6.

A one-year-old Mexican boy who presented severe HF and DCM was properly diagnosed based on the current classification [13]. His parents and brother were apparently healthy individuals with no reported clinical history of SCD syndromes nor cardiac-related deaths within their family. The parents reported that the symptoms in the patient began during the first months of life but had rapidly intensified in the following weeks. The patient was diaphoretic, tachycardic, tachypneic, and hypotensive due to inadequate cardiac output. Palpation of the precordium demonstrated an apical impulse displaced downward and laterally a systolic murmur Grade III/VI and an S3-S4 gallop rhythm were audible in all the cardiac auscultatory focus. The twelve-lead electrocardiogram (Figure 1) showed sinus tachycardia of 159 bpm, left increased precordial lead voltages secondary to ventricular dilation, an incomplete bundle branch left ventricle (BBLV) was apparent and abnormalities in the repolarization were noticed with inverted *T* waves in V3-V6 leads.

**Figure 2:**
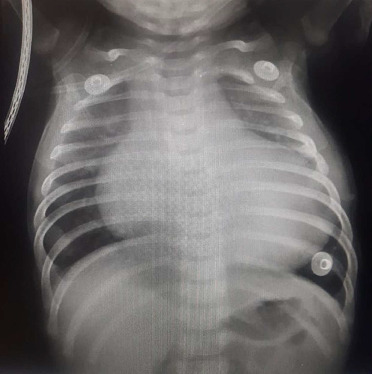
Thoracic roentgenography of the DCM patient. Chest roentgenography demonstrates marked cardiomegaly, pulmonary venous congestion, and pulmonary edema.

Chest roentgenography (Figure 2) demonstrated marked cardiomegaly. The cardiothymic silhouette was enlarged mainly because of left atrial and ventricular dilation. Pulmonary venous congestion and pulmonary edema were evident as well.

**Figure 3:**
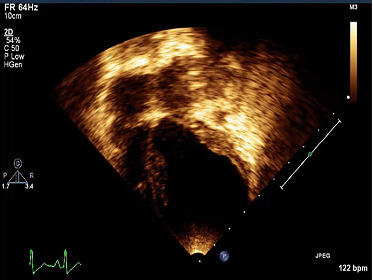
Doppler echocardiography reveals a severe DCM in the proband. Four chambers' view of the echocardiography allows to appreciate the left auricle and the left ventricle severely enlarged and the thinned interventricular septum bulging into the right ventricle.

Echocardiography (Figure 3) confirmed the diagnosis of DCM with the left auricle and LV severely enlarged. End-diastolic and end-systolic LV diameters were increased (diameter at the end of LV diastole of 34 mm; *z* score +5.5, LV diameter at the end of systole of 31 mm; *z* score +7.1). The interventricular septum was bulging into the right ventricle, and the interventricular septum and free wall of the LV appeared diminished. Systolic function was decreased, and LV ejection fraction (LVEF) was calculated at 7%. The origin of both coronary arteries presented normally.

The initial treatment of the patient was based on intravenous diuretics with combined inotropic and vasodilator support (levosimendan and milrinone) with incremental titration as the clinical situation dictated. Once the clinical situation improved and the patient was able to eat, the use of oral beta-blocking agent with bisoprolol was also initiated with incremental titration. In addition to optimal pharmacological management for the HF, with the function of the right ventricle still preserved, the patient underwent surgery for a pulmonary artery band as a bridge therapy for HT based on a recent worldwide experience report [14]. Temporary improvement was obtained, and the patient was sent home with ongoing consultations; however, the child died suddenly at home 6 months later waiting for HT.

### 3.2. Genetic, Bioinformatic, and Conservative Analysis Identified Two Candidate Pathogenic Variants Related to DCM

**Figure 4:**
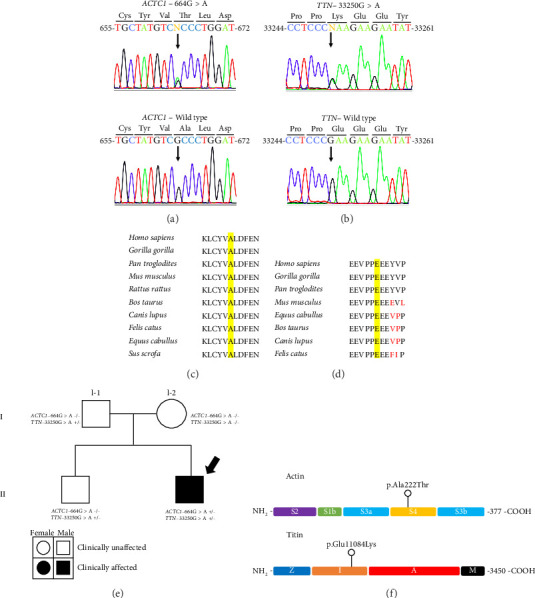
Analysis of confirmed variants in the infant DCM case support their probable pathogenic implication. (a) DNA chromatograms contrast of mutated and native ACTC1 show a heterozygous A > G transition at nucleotide 664, which is traduced into an Ala > Thr substitute ion at 222nd residue in actin protein. (b) DNA chromatograms show a heterozygous 33,250 mutated site of *TTN* from A to G, which is traduced into a Glu > Lys substitution at 11,084 residue in titin protein. (c) Amino acidic alignment of orthologous actin proteins shows that alanine at Position 222 is highly conserved among several species. (d) Amino acidic alignment of orthologous titin proteins shows that glutamic acid at Position 11,084 is well conserved among species. (e) The pedigree chart of the family shows a segregation of *TTN* variant from the father to the proband and his brother, but *ACTC1* variant was spontaneously acquired since it was not present in the parents. The black arrow indicates the proband. (f) Schematic representation of the protein location within the actin and titin protein structures. Actin mutation is located inside the fourth subunit, while titin mutant amino acid is within a region located in the *I* zone of cardiomyocytes' sarcomere. The scheme of actin protein was constructed according to Douglas et al. [15], and titin protein scheme was adapted from Herman et al.'s study [16].

The NGS analysis in the patient suffering DCM yielded 243 Mb of read bases from which 95.61% obtained at least a Q20 score. 99.1% of the reads were on target, and 97.99% of them read at least 20x more with mean sequencing depth of 593 reads *per* amplicon. A summary of the coverage analysis data of the NGS is described in the Supporting Table S1. Alignment and variant calling showed a total of 343 variants, derived only from the 48 structure-related gene panel. After the applied internal filtering criteria, two variant genes were discovered with a heterozygous single-nucleotide variation (SNV) each, whose minor allele frequency (MAF) was cero in the control cohort. Thus, we confirmed the coexpression of a *TTN* (NM_001256850.1:c.33250G > A/NP_001243779.1:p.Glu11084Lys) and *ACTC1* (NM_005159.5:c.664G > A/NP_005150.1:p.Ala222Thr) mutants (Figures 4(a) and 4(b)).

*In silico* predictors suggested the mutations to be deleterious to a certain extent (Table 1). All of them classified the *ACTC1* variant as disease-causing or damaging, while the *TTN* mutation was denoted by PolyPhen-2 as possibly damaging. Additionally, the conservative analysis revealed that both mutated amino-acid residues were situated in well-conserved domains among many orthologous proteins (Figures 4(c) and 4(d)), suggesting the significance of the affected protein sites throughout evolution. Interestingly, no mutation in the 84 electrical-gene panel related to cardiac anomalies was found.

### 3.3. Hereditary Analysis Revealed a Segregated *TTN* Mutation and a *De Novo ACTC1* Mutation

Sanger test validation of the variants and further genealogy analysis conveyed that the *TTN* variant was transmitted from the father, which was also acquired by the proband's sibling. However, to our surprise, the *ACTC1* variant was not present in the parents nor in the brother (Figure 4(e)). As the frequency of spontaneous mutations in human DNA is relatively low due to the DNA polymerase exonuclease activity, we decided to avoid false-positive results by performing Sanger sequencing of the same *loci* again in the four individuals, which resulted in no sequence changes, validating the presence of both mutations.

### 3.4. Computational 3D Modeling Analysis Supports Structural Changes in Actin p.Ala222Thr and Titin p.Glu11084Lys Variants

**Figure 5:**
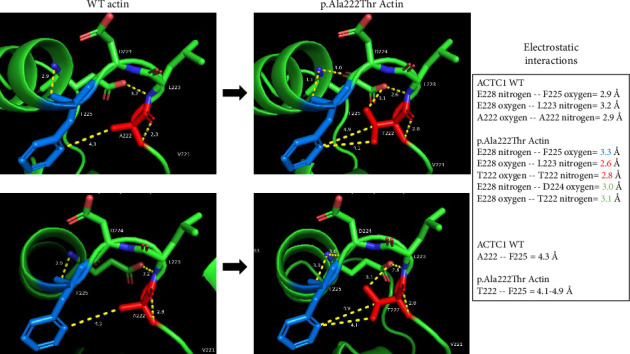
Comparative analysis between computational structures of wild-type (WT) and p.Ala222Thr actins shows changes in some electrostatic interactions shifts. Electrostatic interactions in WT actin neighboring are affected in mutant variant (right images). The most remarkable interaction shifts are specified with yellow dotted lines: E228 nitrogen–F225 oxygen changes from 2.9 Å to 3.3 Å; E228 oxygen–L223 nitrogen changes from to 3.2 Å to 2.6 Å; T222 oxygen–T222 nitrogen changes from 2.9 Å to 2.8 Å; a new hydrogen bond is generated between E228 nitrogen and D224 oxygen (3.0 Å); a new hydrogen bond is formed between the mutated T222 nitrogen and E228 oxygen (3.1 Å). Blue numbers inside the box of electrostatic interactions express an increase in distance compared to the normal state of WT protein, red numbers express a decrease in distance, and green numbers declare new interactions.

Quality parameters declared a reliable template for protein 3D modeling (GMQE = 0.99; identity = 98.67%), and the structure of the monomeric actin was successfully constructed. Among several other observed structural shifts, distance changes between Ala222Thr neighboring residues in subdomain four were observed, as well as hydrogen bond rearrangements including a new steric contact between the mutant substitute 222Thr and 224D. The most relevant changes are depicted and described in Figure 5.

**Figure 6:**
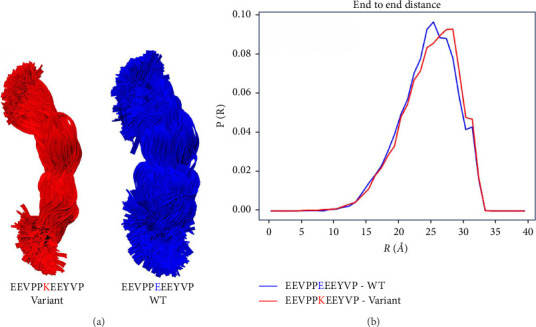
Comparative conformations of 11-residue peptide TTN wild-type show a more compacted state compared to E11084K variant. (a) Spatial distribution of TTN in the sarcomere and modeling of the region around the E11084K mutant. Overlay of all allowed conformations (1300 for each; each ribbon shows a single conformation) of the wild-type (right, blue), and mutant (left, red) 11-mer peptides centered on the E11084K mutant shows that the wild-type can adopt more compact conformations than the mutant. (b) Plot of the probability distribution for the end-to-end length of each peptide shows that the mutation causes an increase in end-to-end distance. Wild-type = EEVPPEEEYVP and mutant = EEVPPKEEYVP.

In the modeling of the region around the amino acid change in titin, this E11084K mutation appears to hinder the formation of compact states, leading to a larger average end-to-end distance for the peptide (Figure 6), which may impact the spatial distribution in the sarcomere.

## 4. Discussion

The presence of genetic deleterious variants expressed in cardiac tissues can lead to structural heart defects. Congenital heart defects compel people susceptible to an unexpected stoppage of the heart by triggering arrhythmias, the principal cause of SCD [17]. These SCD syndromes are mainly explained by the alteration of two groups of cardiac proteins: (1) those that principally constitute the scaffolding of cardiomyocytes and give them support and structure and (2) those related to the action potential and electrical transduction.

In a previous study carried out at the cardiomyopathies and arrhythmias research laboratory of The Children's Hospital of Mexico Federico Gómez, DCM was the most frequent variety of cardiomyopathy (62.5%) and the most common disease of all the hereditary SCD syndromes (including cardiomyopathies and hereditary electrical diseases; 42.37%) [18]. The infant presented in this study corresponds to the case with the most malignant presentation (early diagnosis and death) within this cohort of Mexican children from 0 to 18 years with hereditary SCD syndromes, which give support to the clinical significance of the findings described here.

To date, variants in over 50 genes have been linked to familial DCM [19], unfortunately, many of these cases remain without a clear molecular etiology. Next generation sequencing studies performed by our research team showed that the infant case described here did not possess any genetic variations within a selected group of 87 ion channel genes related to cardiac anomalies (unpublished data). Nonetheless, we found two interesting germline variants within the structural cardiac genes *ACTC1* and *TTN*, which aid in facilitating the identification of possible DCM-causing genes.

The quality of the data generated though NGS is an important parameter to be considered when used to correlate with clinical diagnosis because it is a mark of the experiment's reliability, also giving an insight about the quality of the technical performance of the experiment. Our results pertain to the recommended sequencing depth (120x) for the diagnosis of genetic variants with clinical significance and the detection of heterozygous SNV (35x) [20, 21]. Moreover, the obtained on-target reads percentage and mapped reads surpassed the threshold of a good-quality library (80% and 100,000, respectively) [20]. Other studies searching for new genetic variants linked to cardiomyopathies by NGS showed similar or fewer quality data points in terms of the abovementioned parameters [8, 9, 22–24]. The first genetic mutation related to DCM was identified in *ACTC1* (gene encoding for cardiac alpha actin protein) which was segregated in an autosomal-dominant pattern [25]. Since then, several *ACTC1* mutations have been identified from which up to 20% can cause DCM [26], some of them even overlap with different types of cardiac hereditary diseases [26–30].

Cardiac actin is a fundamental protein located in the sarcomere of cardiomyocytes, and it is crucial for myocardium contraction, force generation, and blood pumping. For this reason, it was very interesting to find that the *ACTC1* mutation in the subject of study was not segregated by either of the parents, denoting its *de novo* origin, a very rare type of “spontaneous” acquisition. To the best of our knowledge, there is no peer-reviewed data regarding a phenotypic association of c.664G > A *ACTC1*/p.Ala222Thr variant in the available literature or in databases such as PubMed, and it is absent in control cohorts as 1000 genomes, exome sequencing project, the exome aggregation consortium (ExAC), or the genome aggregation database (gnomAD). Notwithstanding, it is of important notice that there are five previous reports of this variant from different submitters in ClinVar database, two of them being classified as likely pathogenic in DCM cases also with a *de novo* origin. Based on the American College of Medical Genetics and Genomics (ACMG) guidelines [31] and the features we found, our research team also classified the c.664G > A *ACTC1* variant as likely pathogenic in the present case (categories PM2, PM6, PP3, and PP5 are met). As nonrelated cases, these findings could remark the potential pathogenicity of the variant.

The origin of spontaneous SNV can happen during embryonic development through different mechanisms, and it is estimated that up to 74 SNV can be found in a single germinal genome [32]. The detection of *de novo* variations is frequently initiated by genetic research, as there are DNA changes that do not come from family inheritance. These *de novo* mutations are generally very important in the etiology of diseases because they have been occurring outside of the usual parameters of natural selection, so they are more likely to cause undesirable functional consequences [33]. This strongly suggests that *ACTC1* mutation may play a pathological role in the studied DCM case.

The amino acid change in cardiac actin occurred in a highly conserved region of actin in a variety of species (Figure 4(c)) which constitutes a hallmark that connote its possible pathogenic effect. This actin protein variant has a change from alanine to threonine in the 222nd position, in relation to the human genome reference GRCh37.p7. According to X-ray fiber diffraction studies, this position is located in an intra-subdomain four region [34] (Figure 4(f)). Mutations in Subdomains 1 and 4 of actin have been recognized to likely affect protein-protein interactions [35]. Subdomain 4 of actin maintains electrostatic interactions with proteins such as tropomyosin [15]. Tropomyosin is a key protein in cardiomyocyte contraction because it acts together with troponin to sterically hamper the union of myosin heads when Ca^++^ levels are low [36]. On the other hand, it is scientifically recognized that hydrophilic amino acids of a given polypeptide chain are facing the surface of the molecule in protein folding, while the hydrophobic amino acids are located interiorly. The p.Ala222Thr actin variant provokes exactly the opposite, which could explain our in silico findings where structure and hydrogen bond arrangements change intra-subdomain four. That perhaps leads to defects of actin polymerization in vivo, hampering the formation of filamentous actin and in turn, and the contraction of cardiomyocytes as it was demonstrated for the p.Gly247Asp actin variant, located also in subdomain four [37]. Either conformational or affinity changes between tropomyosin and actin or defects of actin polymerization would lead to cardiac remodeling such as DCM. However, further specific functional studies are needed to corroborate this hypothesis.

The index case described here also had a cosegregation of a heterozygous mutation in the *TTN* gene that encodes titin protein. For a matter of fact, its crucial role and its nucleotide length make *TTN* the major affected gene in DCM cases [38, 39]. Titin is located in the sarcomeres, between actin and myosin filaments, and it is the longest protein in humans. Its main purpose is to provide cell elasticity and structural network organization, preventing damage caused by cardiomyocyte resistance to contractions and providing passive tension to striated muscle. This ability is mainly attributed to an elastic structure enriched of proline, glutamate, valine, and lysine (PEVK), which constitutes an entropic biological spring and mimics a shock absorber [38, 40]. The conformations of the PEVK region appear to be sensitive to both pH and calcium concentration [41, 42] and, interestingly, the amino acid change in the titin variant that our research group found was a substitution of acidic and negatively charged glutamic acid by an alkaline and positive charged lysine in Position 11,084, which is also a conserved residue among species (Figure 4(d)). Specifically, this amino acid position is within the distal I band according to the spatial distribution of *TTN* in the sarcomere (Figure 4(f)) region where truncating variants have already been associated with DCM [16, 38, 43].

However, despite the efforts of the scientific community to assess the real implications of missense *TTN* variants, the majority of them are still considered ambiguous because it is hard (and sometimes contradictory) to determine their pathogenicity with current approaches, leaving them without both molecular and a precise clinical significance [44]; functional studies are also difficult to carry out due in part to the nature of titin, which is considered the largest coded protein in humans. In the present study, the c.33250G > A *TTN* allele is considered as a variant of uncertain significance (VUS) according to the ACMG guidelines because of the contradictory features we found when analyzing the data in the giving clinical and scientific context.

The mechanism by which the E11084K mutant contributes to cardiac disease may plausibly be one or more of several related effects. Charge pairs direct formation of compact configurations of the titin chain upon release of tension [45], so the insertion of a positive charge farther to the *N*-terminus of the PEVK region may disrupt the formation and stability of the compact or folded states that normally contribute to avoid mechanical damage. Disruption of the ability of titin to extend and collapse may inhibit its mechanosensory mechanism. On the other hand, it is possible that the poly-*E* motifs in titin interacts with *F*-actin [46], affecting the interactions that mediate the mechanical memory of the sarcomere.

Previous reports of this *TTN* variant are currently found in ClinVar database in the clinical context of DCM and other myopathies, six of them being classified as VUS, and two as benign. On the other hand, some observations explain the pathological implications that some missense *TTN* variants can represent in some individuals, as the one that demonstrated to cause partial protein unfolding and domain destabilization [47]. Also, an in vitro study revealed that induced pluripotent stem cell cardiomyocytes carrying a missense mutation within the sarcomere *Z/I* junction portion in titin developed a DCM-like phenotype [48]. Nonetheless, it is of noticeable importance that the missense c.33250G > A variant addressed in the present study was observed to be inherited by the father of the study infant subject, which was also acquired by his brother but who did not present any cardiac symptomatology at the time of sampling, suggesting a minor cardiac pathogenic effect when expressed alone.

Due to the suggestive pathogenic elements in all the previous examined data, one hypothesis to be considered in this work regarding the onset of DCM in our patient is an autosomal dominant behavior of *ACTC1* mutation. Notwithstanding, due to the high complexity of cardiomyopathies, it is also suggestive that the cause of the malignant condition reported here may be derived from a polygenic synergistic effect of both *ACTC1* and *TTN* variants, where this double hit is necessary to impair the phenotypic manifestation of an early and severe DCM. This type of polygenic effect has been also described and demonstrated in vitro and in vivo in another similar case of DCM [49], where two separate variants in *VLN* and *TPM1* could not be associated with the DCM phenotype when evaluated separately but together, combined with disease-related stress factors.

A limitation in our study is that we may be missing other factors that can prompt DCM onset, such as environmental challenges, variants with epigenetic significance, genes in other networks that either directly or indirectly interact with the sequenced structural genes, or rare somatic mutations. Altogether, as many other VUS, it is necessary to go further with this study and to carry out functional experiments to determine whether these two rare variants can trigger DCM and the possible mechanisms involved. Finally, by comparing our results with the available literature, we can conclude there is a diverse genetic background in DCM patients. Our report of the germline variants in *TTN* and *ACTC1* connote the complexity of the possible molecular causes of SCD syndromes and affirm that there are still pathogenic genetic factors we could be facing for the first time, which challenges the prescription of the more appropriate and beneficial treatment.

## Figures and Tables

**Table 1:** *In silico* predictors indicate the level of probable pathogenicity of the *ACTC1* and *TTN* mutations.

Gene (protein)	Locus (GRCh37)	DNA change	Amino acid change	PolyPhen-2 (score)	PROVEAN (score)	SIFT (score)
ACTC1^a^ (cardiac alpha actin)	chr15:35084435	c.664G > *A*	p.Ala222Thr	Probably damaging (0.964)	Deleterious (−2.99)	Damaging (0)
TTN^b^ (titin)	chr2:179542438	c.33250G > *A*	p.Glu11084Lys	Possibly damaging (0.892)	Neutral (−1.61)	Tolerated (0.33)

^a^ACTC1 reference sequence NM_005159.5.

^b^TTN reference sequence NM_001256850.1.

## Data Availability

The datasets supporting the current study have not been deposited in a public repository because these data are available by the PI and are protected by HIPPA regulation under the LIMR/MLH hospital. They are available in a spreadsheet from NGS assays in Lankenau Institute for Medical Research, Wynnewood, Pennsylvania, the United States of America. Raw and other data are available upon request from the authors.

## Supporting Information

Supporting Information 1 Table S1: Coverage analysis of DCM patient targeted sequencing. Technical description of the data derived from sequencing, which evaluates experiment's good quality and reliability in terms of the nucleotide sequence accuracy.

Supporting Information 2 Table S2: Analyzed panel of 48 genes with structural cardiac importance in the proband. Compilation of the sequenced structure-related cardiac genes transcripts included in one of the panels.

Supporting Information 3 Table S3: Analyzed panel of 73 genes with electrical cardiac importance in the proband. Compilation of the sequenced electrical-related cardiac genes transcripts included in one of the panels.
