# Supplementary material for: A De Novo Mutation in ACTC1 and a TTN Variant Linked to a Severe Sporadic Infant Dilated Cardiomyopathy Case
Source: Case Rep Genet. 2024 Dec 28;2024:9517735. doi: 10.1155/crig/9517735 (PMC11699985; doi:10.1155/crig/9517735)
Supplement: Supporting Information 1 — Table S1: Coverage analysis of DCM patient targeted sequencing. Technical description of the data derived from sequencing, which evaluates experiment's good quality and reliability in terms of the nucleotide sequence accuracy. [file 9517735.f1.docx]

**Supplementary material: Table S1. Coverage analysis of DCM patient targeted sequencing.** Technical description of the data derived from sequencing, which evaluates experiment’s good quality and reliability in terms of the nucleotide sequence accuracy.

| **Read bases (bp)** | **≥Q20 (%pb)** | **Total reads (*ISP*)** | **Mapped reads (*ISP*)** | **On target reads (%)** | **Mean sequencing depth** | **Mean read length** | **1x (%)** | **20x (%)** | **100x (%)** |
| --- | --- | --- | --- | --- | --- | --- | --- | --- | --- |
| 242,994,799 | 232,348,958 (95.61%) | 1,282,571 | 1,281,155 | 99.10 | 593.0 | 189 | 99.77 | 97.99 | 95.19 |

bp= base pairs; ≥Q20= Phred Q score indicating at least 99% chance of assigning the correct base; ISP= Ionic Sphere Particles; 1x= percentage of amplicons covered at least once; 20x= percentage of amplicons covered at least twenty times; 100x= percentage of amplicons covered at least one hundred times
