# Supplementary material for: A De Novo Mutation in ACTC1 and a TTN Variant Linked to a Severe Sporadic Infant Dilated Cardiomyopathy Case
Source: Case Rep Genet. 2024 Dec 28;2024:9517735. doi: 10.1155/crig/9517735 (PMC11699985; doi:10.1155/crig/9517735)
Supplement: Supporting Information 2 — Table S2: Analyzed panel of 48 genes with structural cardiac importance in the proband. Compilation of the sequenced structure-related cardiac genes transcripts included in one of the panels. [file 9517735.f2.docx]

**Table S2. Analyzed panel of 48 genes with structural cardiac importance in the proband.** Compilation of the sequenced structure-related cardiac genes transcripts included in one of the panels.

| **Gene** | **Genomic location** | **Reference sequence** | **Gene** | **Genomic location** | **Reference sequence** |
| --- | --- | --- | --- | --- | --- |
| *ACTC1_a_* | 15q14 | [NM_005159.5](https://www.ncbi.nlm.nih.gov/nuccore/NM_005159.5) | *MYH7 _a_* | 14q11.2 | [NM_000257.4](https://www.ncbi.nlm.nih.gov/nuccore/NM_000257.4) |
| *ACTN2_a_* | 1q43 | NM_001103.4 | *MYL2* | 12q24.11 | [NM_000432.4](https://www.ncbi.nlm.nih.gov/nuccore/NM_000432.4) |
| *ANKRD1 _a_* | 10q23.31 | [NM_014391.3](https://www.ncbi.nlm.nih.gov/nuccore/NM_014391.3) | *MYL3* | 3p21.31 | [NM_000258.3](https://www.ncbi.nlm.nih.gov/nuccore/NM_000258.3) |
| *BAG3 _a_* | 10q26.11 | [NM_004281.4](https://www.ncbi.nlm.nih.gov/nuccore/NM_004281.4) | *MYLK2* | 20q11.21 | [NM_033118.4](https://www.ncbi.nlm.nih.gov/nuccore/NM_033118.4) |
| *CRYAB _a_* | 11q23.1 | [NM_001289807.1](https://www.ncbi.nlm.nih.gov/nuccore/NM_001289807.1) | *MYOZ2* | 4q26 | [NM_016599.5](https://www.ncbi.nlm.nih.gov/nuccore/NM_016599.5) |
| *CSRP3 _a_* | 11p15.1 | [NM_003476.5](https://www.ncbi.nlm.nih.gov/nuccore/NM_003476.5) | *MYPN _a_* | 10q21.3 | NM_001256267.1 |
| *DES _a_* | 2q35 | NM_001927.4 | *NEBL* | 10p12.31 | NM_006393.3 |
| *DMD _a_* | Xp21.2-p21.1 | NM_004006.3 | *NEXN _a_* | 1p31.1 | NM_144573.4 |
| *DSC2* | 18q12.1 | NM_024422.6 | *PKP2* | 12p11.21 | NM_004572.3 |
| *DSG2 _a_* | 18q12.1 | [NM_001943.5](https://www.ncbi.nlm.nih.gov/nuccore/NM_001943.5) | *PLN _a_* | 6q22.31 | [NM_002667.5](https://www.ncbi.nlm.nih.gov/nuccore/NM_002667.5) |
| *DSP _a_* | 6p24.3 | [NM_004415.4](https://www.ncbi.nlm.nih.gov/nuccore/NM_004415.4) | *PSEN1 _a_* | 14q24.2 | NM_000021.4 |
| *DTNA* | 18q12.1 | NM_001390.4 | *PSEN2 _a_* | 1q42.13 | NM_000447.3 |
| *EMD* | Xq28 | [NM_000117.3](https://www.ncbi.nlm.nih.gov/nuccore/NM_000117.3) | *RBM20 _a_* | 10q25.2 | [NM_001134363.3](https://www.ncbi.nlm.nih.gov/nuccore/NM_001134363.3) |
| *EYA4 _a_* | 6q23.2 | NM_172105.3 | *SGCD _a_* | 5q33.2-q33.3 | NM_001128209.2 |
| *FHL1 _a_* | Xq26.3 | NM_001159702.3 | *TAZ _a_* | Xq28 | NM_000116.5 |
| *GATAD1 _a_* | 7q21.2 | [NM_021167.5](https://www.ncbi.nlm.nih.gov/nuccore/NM_021167.5) | *TCAP* | 17q12 | [NM_003673.4](https://www.ncbi.nlm.nih.gov/nuccore/NM_003673.4) |
| *GLA* | Xq22.1 | [NM_000169.3](https://www.ncbi.nlm.nih.gov/nuccore/NM_000169.3) | *TMEM43* | 3p25.1 | [NM_024334.3](https://www.ncbi.nlm.nih.gov/nuccore/NM_024334.3) |
| *JUP* | 17q21.2 | NM_002230.4 | *TMPO _a_* | 12q23.1 | NM_003276.2 |
| *LAMA4 _a_* | 6q21 | NM_001105206.3 | *TNNC1* | 3p21.1 | [NM_003280.3](https://www.ncbi.nlm.nih.gov/nuccore/NM_003280.3) |
| *LAMP2* | Xq24 | NM_001122606.1 | *TNNI3 _a_* | 19q13.42 | [NM_000363.5](https://www.ncbi.nlm.nih.gov/nuccore/NM_000363.5) |
| *LDB3 _a_* | 10q22.2 | NM_007078.3 | *TNNT2 _a_* | 1q32.1 | NM_001276345.1 |
| *LMNA _a_* | 1q22 | NM_170707.4 | *TPM1 _a_* | 15q22.2 | NM_001018020.2 |
| *MYBPC3 _a_* | 11p11.2 | [NM_000256.3](https://www.ncbi.nlm.nih.gov/nuccore/NM_000256.3) | *TTN _a_* | 2q31.2 | NM_001256850.1 |
| *MYH6 _a_* | 14q11.2 | [NM_002471.4](https://www.ncbi.nlm.nih.gov/nuccore/NM_002471.4) | *VCL _a_* | 10q22.2 | NM_014000.3 |

*_a_* Known DCM susceptible genes.
