# Supplementary material for: A De Novo Mutation in ACTC1 and a TTN Variant Linked to a Severe Sporadic Infant Dilated Cardiomyopathy Case
Source: Case Rep Genet. 2024 Dec 28;2024:9517735. doi: 10.1155/crig/9517735 (PMC11699985; doi:10.1155/crig/9517735)
Supplement: Supporting Information 3 — Table S3: Analyzed panel of 73 genes with electrical cardiac importance in the proband. Compilation of the sequenced electrical-related cardiac genes transcripts included in one of the panels. [file 9517735.f3.docx]

**Table S3. Analyzed panel of 73 genes with electrical cardiac importance in the proband.** Compilation of the sequenced electrical-related cardiac genes transcripts included in one of the panels.

| **Gene** | **Genomic location** | **Reference sequence** | **Gene** | **Genomic location** | **Reference sequence** |
| --- | --- | --- | --- | --- | --- |
| *ABCC8* | 11p15.1 | NM_001351297.2 | *KCND3* | 1p13.2 | NM_001378969.1 |
| *ABCC9* | 12p12.1 | NM_020297.4 | *KCNE1* | 21q22.12 | NM_000219.6 |
| *AGTR1* | 3q24 | NM_000685.5 | *KCNE2* | 21q22.11 | NM_172201.2 |
| *AKAP9* | 7q21.2 | NM_005751.5 | *KCNE3* | 11q13.4 | NM_005472.5 |
| *ANK2* | 4q25-q26 | NM_001148.601927.4 | *KCNE4* | 2q36.1 | NM_080671.4 |
| *CACNA1C* | 12p13.33 | NM_001129842.2 | *KCNE5* | Xq23 | NM_012282.4 |
| *CACNA2D1* | 7q21.11 | NM NM_000722.4 | *KCNH2* | 7q36.1 | NM_000238.4 |
| *CACNA2D2* | 3p21.31 | [NM_006030.4](https://www.ncbi.nlm.nih.gov/nuccore/NM_001943.5) | *KCNIP2* | 10q24.32 | NM_173191.3 |
| *CACNB1* | 17q12 | NM_000723.5 | *KCNJ2* | 17q24.3 | NM_000891.3 |
| *CACNB2* | 10p12.33-p12.31 | NM_201596.3 | *KCNJ8* | 12p12.1 | NM_004982.4 |
| *CACNB3* | 12q13.12 | NM_000725.4 | *KCNJ9* | 1q23.2 | NM_004983.3 |
| *CACNB4* | 2q23.3 | NM_000726.5 | *KCNJ10* | 1q23.2 | NM_002241.5 |
| *CACNG4* | 17q24.2 | NM_014405.4 | *KCNQ1* | 11p15.5-p15.4 | NM_000218.3 |
| *CACNG5* | 17q24.2 | NM_145811.3 | *KCNQ2* | 20q13.33 | NM_172107.4 |
| *CACNG6* | 19q13.42 | NM_145814.2 | *PRKAG2* | 7q36.1 | NM_016203.4 |
| *CALM1* | 14q32.11 | NM_006888.6 | *PXDNL* | 8q11.22-q11.23 | NM_144651.5 |
| *CALM2* | 2p21 | NM_001743.6 | *RYR2* | 1q43 | NM_001035.3 |
| *CASQ2* | 1p13.1 | NM_001232.4 | *SCN1A* | 2q24.3 | NM_001165963.4 |
| *CAV1* | 7q31.2 | NM_001753.5 | *SCN2A* | 2q24.3 | NM_001040142.2 |
| *CAV2* | 7q31.2 | NM_001233.5 | *SCN3A* | 2q24.3 | NM_006922.4 |
| *CAV3* | 3p25.3 | NM_033337.3 | *SCN4A* | 17q23.3 | NM_000334.4 |
| *DPP6* | 7q36.2 | NM_130797.4 | *SCN5A* | 3p22.2 | NM_000335.5 |
| *DPP7* | 9q34.3 | NM_013379.3 | *SCN7A* | 2q24.3 | NM_002976.4 |
| *DPP8* | 15q22.31 | NM_130434.5 | *SCN10A* | 3p22.2 | NM_006514.4 |
| *DPP9* | 19p13.3 | NM_139159.5 | *SCN1B* | 19q13.11 | NM_001037.5 |
| *DPP10* | 2q14.1 | NM_020868.6 | *SCN2B* | 11q23.3 | NM_004588.5 |
| *FGF12* | 3q28-q29 | NM_004113.6 | *SCN3B* | 11q24.1 | NM_001040151.2 |
| *FGF13* | Xq26.3-q27.1 | NM_004114.5 | *SCN4B* | 11q23.3 | NM_174934.4 |
| *GJA5* | 1q21.2 | NM_181703.4 | *SEMA3C* | 7q21.11 | NM_006379.5 |
| *GPD1L* | 3p22.3 | NM_015141.4 | *SNTA1* | 20q11.21 | NM_003098.3 |
| *HCN2* | 19p13.3 | NM_001194.4 | *SUR1* | 11p15.1 | NM_001287174.3 |
| *HCN4* | 15q24.1 | NM_005477.3 | *SUR2A* | 12p12.1 | NM_005691.4 |
| *HEY2* | 6q22.31 | NM_012259.3 | *TBX5* | 12q24.21 | NM_000192.3 |
| *IRX3* | 16q12.2 | NM_024336.3 | *TRPM4* | 19q13.33 | NM_017636.4 |
| *IRX4* | 5p15.33 | NM_016358.3 |  |  |  |
| *IRX5* | 16q12.2 | NM_005853.6 |  |  |  |
| *JPH2* | 20q13.12 | NM_020433.5 |  |  |  |
| *KCNA4* | 11p14.1 | NM_002233.4 |  |  |  |
| *KCNA5* | 12p13.32 | NM_002234.4 |  |  |  |
| *KCNN1* | 19p13.11 | [NM_001386974.1](https://www.ncbi.nlm.nih.gov/nuccore/NM_000257.4) |  |  |  |
| *KCNN2* | 5q22.3 | [NM_021614.4](https://www.ncbi.nlm.nih.gov/nuccore/NM_000432.4) |  |  |  |
| *KCNN3* | 1q21.3 | [NM_002249.6](https://www.ncbi.nlm.nih.gov/nuccore/NM_000258.3) |  |  |  |
| *KCNK1* | 1q42.2 | NM_002245.4 |  |  |  |
| *KCNK2* | 1q41 | [NM_001017425.3](https://www.ncbi.nlm.nih.gov/nuccore/NM_016599.5) |  |  |  |
| *KCNK3* | 2p23.3 | NM_002246.3 |  |  |  |
